# Supplementary material for: Early warning of some notifiable infectious diseases in China by the artificial neural network
Source: R Soc Open Sci. 2020 Feb 19;7(2):191420. doi: 10.1098/rsos.191420 (PMC7062078; doi:10.1098/rsos.191420)
Supplement: Supplementary material of early warning of some notifiable infectious diseases in China by the artificial neural network [file rsos191420supp1.docx]

*Royal Society Open Science*

**Supplemental material for early warning of some notifiable infectious diseases in China by the artificial neural network**

Zuiyuan Guo, Kevin He, Dan Xiao

The calculation process of the model is described in detail below:

**Real-time recurrent learning**

The learning algorithm for real-time recurrent learning (RTRL) refers to real-time adjustment of the synaptic weight of the fully connected network. Figure 1A shows the structural layout of the real-time recurrent network, which is composed of $q$ neurons and $m$ external inputs. The network has two different layers: the concatenated input-feedback layer and the processing layer for calculating nodes; the former is expressed as blue squares, and the latter is presented as green circles. The output vector $\mathbf{x}_{\mathbf{n+1}}$ in the $n+1$^th^ time step is written in the following extended form:

(1)

where all neurons are presumed to have the same activation function . The $(q+m+1)\times1$ vector $\mathbf{w}_{j}$ is the synaptic weight vector of neuron $j$ in the recurrent network:

$\mathbf{w}_{j}\mathbf{=}\left[\begin{matrix} \mathbf{w}_{a,j} \\ \mathbf{w}_{b,j} \end{matrix}\right]$ $j=1,2,\cdots,q$ (2)

Matrices $\mathbf{W}_{a}$ and $\mathbf{W}_{b}$ are defined below:

$$\mathbf{W}_{a}=\left[\begin{matrix} w_{1,1} & w_{1,2} & \cdots& w_{1,q} \\ w_{2,1} & w_{2,2} & \cdots& w_{2,q} \\ \vdots& \vdots& \vdots& \vdots\\ w_{q,1} & w_{q,2} & \cdots& w_{q,q} \end{matrix}\right]$$

and

$$\mathbf{W}_{b}=\left[\begin{matrix} b_{1} & w_{1,q+2} & \cdots& w_{1,m+q+1} \\ b_{2} & w_{2,q+2} & \cdots& w_{2,m+q+1} \\ \vdots& \vdots& \vdots& \vdots\\ b_{q} & w_{q,q+2} & \cdots& w_{q,m+q+1} \end{matrix}\right]$$

where $\mathbf{w}_{a,j}$ and $\mathbf{w}_{b,j}$ are the *j*^th^ columns of the transpose matrices $\mathbf{W}_{a}^{T}$ and $\mathbf{W}_{b}^{T}$, respectively. The $(q+m+1)\times1$ vector $\mathbf{\xi}_{n}$ is defined as:

$\mathbf{\xi}_{n}=\left[\begin{matrix} \mathbf{x}_{n} \\ \mathbf{u}_{n} \end{matrix}\right]$ (3)

where $\mathbf{x}_{n}$ is the state vector of $q\times1$ and $\mathbf{u}_{n}$ is the output vector of $(m+1)\times1$. The first element of $\mathbf{u}_{n}$ is +1, the first element of the corresponding $\mathbf{w}_{b,j}$ is the bias $\mathbf{b}_{j}$ used in neuron *j*, and other *m* elements were used as inputs of the epidemic information. Four types of respiratory infectious diseases and 4 types of digestive tract infectious diseases were analyzed. The former category includes measles, influenza, rubella, and mumps, and the latter category includes hepatitis A, hepatitis E, typhoid and paratyphoid fever, and bacterial and amoebic dysentery. Therefore, $m=4$.

The numbers of new confirmed cases per month were used as the input variables, which should be standardized before being introducing into the model. After this process was finished, the average value of all input variables should be approximately 0, no correlation should exist between the variables, and covariances should be approximately equal to ensure that different synaptic weights in the network can be learned at approximately the same speed. We used principal component analysis for standardization. The numbers of new confirmed cases of 4 diseases in one month were used as a 4×1 vector $\mathbf{z}$. We believed that $\mathbf{z}$ conformed to a normal distribution, and the probability density function was:

$f(\mathbf{z})=(2\pi)^{-m/2}\left|\Sigma_{i}\right|^{-\frac{1}{2}}\exp\{-\frac{1}{2}(\mathbf{z}_{i}-\mu_{i})^{T}\Sigma_{i}^{-1}(\mathbf{z}_{i}-\mu_{i})\}$ $i=1,2,\cdots,12$ (4)

where $i$ indicates the month, $\Sigma_{i}$ indicates the covariance matrix (a positive-definite matrix), and $\mathbf{\mu}_{i}$ indicates the vector composed of the average value of the numbers of cases. First, eigenvalue decomposition is performed on $\Sigma_{i}$:

$\Sigma_{i}=\mathbf{P}_{i}\Lambda_{i}\mathbf{P}_{i}^{T}$ (5)

Where $\mathbf{\Lambda}$ indicates the diagonal matrix composed of eigenvalues, and $\mathbf{P}$ indicates the matrix composed of orthogonal standardized feature vectors. $\mathbf{z}_{i}$ was standardized according to different months to obtain the input vector of the network $\mathbf{u}_{i}$:

$\mathbf{u}_{i}=\Lambda_{i}^{-\frac{1}{2}}\mathbf{P}_{i}^{T}(\mathbf{z}_{i}-\mu_{i})$ (6)

To develop a clearer expression, the new matrices $\mathbf{R}_{j,n}$, $\mathbf{U}_{j,n}$, and $\mathbf{\Phi}_{n}$ were introduced and are described below:

1. $\mathbf{R}_{j,n}$ is the $q\times(q+m+1)$ matrix composed of the partial derivatives of

weight $\mathbf{w}_{j}$ in state $\mathbf{x}_{n}$:

$\mathbf{R}_{j,n}=\frac{\partial\mathbf{x}_{n}}{\partial\mathbf{w}_{j}}$ ，$j=1,2,\cdots,q$ (7)

1. $\mathbf{U}_{j,n}$ is the $q\times(q+m+1)$ matrix. Except for the *j*^th^ row, which was equal to

vector $\xi_{n}$, all other rows were $\mathbf{0}$.

$\mathbf{U}_{j,n}=\left[\begin{matrix} \mathbf{0} \\ \mathbf{\xi}_{\mathbf{n}} \\ \mathbf{0} \end{matrix}\right]$ *j*^th^ row (8)

1. $\mathbf{\Phi}_{n}$ is the diagonal matrix of $q\times q$. Its *j*^th^ diagonal element is the partial

derivative of the activation function on its independent variables, which can be written as:

(9)

With these definitions, $\mathbf{w}_{j}$ can be derived using equation (1). The chain rule of calculus was used to obtain the following recurrence formula:

$\mathbf{R}_{j,n+1}=\mathbf{\Phi}_{n}(\mathbf{W}_{a,n}\mathbf{R}_{j,n}+\mathbf{U}_{j,n})$ (10)

This recurrence formula describes the nonlinear state dynamics of the RTRL process, which was the state evolution.

To describe this learning process, matrix $\mathbf{R}_{j,n}$ must be associated with the gradient of the error curve facing $\mathbf{w}_{j}$. Therefore, the $p\times1$ error vector was first defined using the metric equation:

$\mathbf{e}_{n}=\mathbf{d}_{n}-\mathbf{y}_{n}=\mathbf{d}_{n}-\mathbf{x}_{n}^{p}$ (11)

where $p$ is the dimension of the output vector $\mathbf{y}_{n}$, $\mathbf{x}_{n}^{p}$ indicates the vector composed of the last $p$ elements of $\mathbf{x}_{n}$, and $\mathbf{W}_{c}$ indicates the $p\times q$ matrix composed of the last $p$ rows of $\mathbf{W}_{a}$. The synaptic weights of *p* neurons constitute the $p\times(q+m+1)$ matrix. $\mathbf{d}_{n}$ indicates the expected response. The cumulative probabilities of 168 training samples were calculated under a multivariate normal distribution, which was arranged from low to high, and the 90% quartile was used as the threshold value. The values of $\mathbf{d}_{n}$were only 1 and -1, which denote over the threshold value and lower than the threshold value, respectively. The squared instantaneous error sum defined based on $\mathbf{e}_{n}$ was:

$E_{n}=\frac{1}{2}\mathbf{e}_{n}^{T}\mathbf{e}_{n}$ (12)

The error sum was used as the minimized pricing function to find its differential value to the weight vector $\mathbf{w}_{j}$:

$\frac{\partial E_{n}}{\partial\mathbf{w}_{j}}=\left(\frac{\partial\mathbf{e}_{n}^{T}}{\partial\mathbf{w}_{j}}\right)\mathbf{e}_{n}$ (13)

The gradient descent method was used to obtain the optimal solution of the weight vector $\mathbf{w}$. Therefore, it was used in the adjustment of the synaptic weight vector $\mathbf{w}_{j,n}$ of neuron *j*:

$\Delta\mathbf{w}_{j,n}=-\eta_{n}\frac{\partial E_{n}}{\partial\mathbf{w}_{j}}=\eta_{n}(\mathbf{\Phi}_{n}^{p}\mathbf{W}_{c}\mathbf{R}_{j,n})^{T}\mathbf{e}_{n}$ (14)

where $\eta_{n}$ was the learning rate parameter, , and $\mathbf{R}_{j,n}$ was determined by formula (7). Because $p=1$ in the model, $\mathbf{W}_{c}=\mathbf{w}_{a,q}^{T}$. The activation function used the sigmoid nonlinear equation in the form of the hyperbolic tangent function.

$$\upsilon_{j,n}=\mathbf{w}_{j}^{T}\mathbf{\xi}_{n}$$

(15)

where $\upsilon_{j,n}$ is the induced local field of neuron *j*, and $x_{j,n+1}$ is the state of the

activation function of neuron *j* at the *n*+1 moment. Here, the appropriate values of *a*

and *b* were specified as:

$$a=1.7159$$

and

$$b=\frac{2}{3}$$

because such values allowed the sigmoid function to have the following

good properties:

1) and .

2) The gradient of at the original point is approximately equal to 1:

3) The expected response $\mathbf{d}_{n}$ is +1 or -1.

The specific calculation is shown in Table 1.

Table 1. Summary of implementation of the recurrent learning algorithm

Parameter:

m = input space dimension

q = state space dimension

p = output space dimension

$w_{j}=$synaptic weight vector of neuron *j*,$j=1,2,\cdots,q$.

Initialization:

1. The initial value of the synaptic weight was assigned a smaller value selected

from the uniform distribution.

1. The state vector was set as $\mathbf{x}(0)=\mathbf{0}$.
2. For $j=1,2,\cdots,q$, $\mathbf{R}_{j,0}=\mathbf{0}$ was set.

Calculation: For $n=0,1,2,\cdots,$ the calculation was:

$$\mathbf{e}_{n}=\mathbf{d}_{n}-\mathbf{W}_{c}\mathbf{x}_{n}$$

$$\mathbf{w}_{j,n+1}=\mathbf{w}_{j,n}+\eta_{n}(\mathbf{\Phi}_{n}^{p}\mathbf{W}_{c}\mathbf{R}_{j,n})^{T}\mathbf{e}_{n}$$

$$\mathbf{R}_{j,n+1}=\mathbf{\Phi}_{n}(\mathbf{W}_{a,n}\mathbf{R}_{j,n}+\mathbf{U}_{j,n})$$

**Extended Kalman filter (EKF)**

Using the concept of sequential state estimation and based on the recurrent network structure established in Figure 1A, the EKF was used to train the network. $n$ was used to record every time step in the supervised training of the network, and vector $\mathbf{w}_{n}$ was used to record the entire set of calculated network synaptic weights at time step $n$. The construction method first placed the synaptic weight of the first neuron in the processing layer on the very top followed by the synaptic weight of the second neuron; this method was continued until the last neuron in the processing layer. The network state space model under training was defined using the following model:

1) System (state) model: The model was described using the following random walk:

$\mathbf{w}_{n+1}=\mathbf{w}_{n}+\mathbf{\omega}_{n}$ (16)

The dynamic noise $\mathbf{\omega}_{n}$ was the white Gaussian noise, the average value was $\mathbf{0}$, and the covariance matrix was $\mathbf{Q}_{\omega}$.

2) Measurement model: The model was described using the following equation:

$\mathbf{d}_{n}=\mathbf{b}(\mathbf{w}_{n},\mathbf{v}_{n},\mathbf{u}_{n})+\mathbf{\upsilon}_{n}$ (17)

where new units are defined below:

$\mathbf{d}_{n}$ is the observable expected value, which was consistent with that in the RTRL algorithm.

$\mathbf{v}_{n}$ indicates the vector activated by the recurrent node in the network and shows the internal state. Its elements were listed in the same order as the weight vector $\mathbf{w}_{n}$.

$\mathbf{u}_{n}$ indicates the network input vector after standardization and was the driving force acting in the network.

$\mathbf{\upsilon}_{n}$ indicates the measurement noise vector, which was assumed to be a multivariate noise process, its average value was $\mathbf{0}$, and it had the diagonal covariance matrix $\mathbf{Q}_{\upsilon}$. The vector-valued measurement function obtained from equation (17) was the same as the activation function in RTRL, indicating that the mapping from the input layer to the output layer was nonlinear. It was the only nonlinear source in the recurrent network state space model.

After obtaining the training sample $\{\mathbf{u}_{n},\mathbf{d}_{n}\}_{n=1}^{N}$, we should perform supervised training of the recurrent network using the successive state estimator method. Based on the nonlinearity of the network, the EKF was used to complete this work. Figure 1B shows the basic framework of this algorithm. The state space of the model included two main parts:

1. The innovations process, which was defined as:

$\mathbf{\alpha}_{n}=\mathbf{d}_{n}-\mathbf{b}(\widehat{\mathbf{w}}_{n|n-1},\mathbf{v}_{n},\mathbf{u}_{n})$ (18) where the expected response $\mathbf{d}_{n}$ indicates the observed value of the EKF.

1. The weight (state) update, which was defined as:

$\widehat{\mathbf{w}}_{n|n}=\widehat{\mathbf{w}}_{n|n-1}+\mathbf{G}_{n}\mathbf{\alpha}_{n}$ (19)

where $\widehat{\mathbf{w}}_{n|n-1}$ indicates the predictive estimation of the weight vector $\mathbf{w}$ of the recurrent network at time *n* under the condition of giving the observed value $\mathbf{d}_{n-1}$ at $n-1$. $\widehat{\mathbf{w}}_{n|n}$ is the updated estimation of $\mathbf{w}$ after receiving the observed value $\mathbf{d}_{n}$. Matrix $\mathbf{G}_{n}$ shows the Kalman gain, which was the integral part of the EKF algorithm. The specific calculation process is shown in Table 2.

Table 2. Summary of the EKF algorithm of supervised training of the recurrent network

Training sample:

$$F=\{\mathbf{u}_{n},\mathbf{d}_{n}\}_{n=1}^{N}$$

where $\mathbf{u}_{n}$ is the input vector acting in the recurrent network, and $\mathbf{d}_{n}$is the corresponding expected response.

Parameters and variables of the EKF:

: vector-valued measurement function

$\mathbf{B}$ ：linear measurement matrix

$\mathbf{w}_{n}$：weight vector at the time step *n*

$\widehat{\mathbf{w}}_{n|n-1}$：predictive estimation of the weight vector

$\widehat{\mathbf{w}}_{n|n}$： filtering estimation of the weight vector

$\mathbf{v}_{n}$：vector activated by recurrent nodes

$\mathbf{y}_{n}$：produced output vector corresponding to the input vector $\mathbf{u}_{n}$

$\mathbf{Q}_{\omega}$：covariance matrix of dynamic noise $\omega_{n}$

$\mathbf{Q}_{\upsilon}$：covariance matrix of measurement noise $\mathbf{v}_{n}$

$\mathbf{G}_{n}$：Kalman gain

$\mathbf{P}_{n|n-1}$：predictive error covariance matrix

$\mathbf{P}_{n|n}$：filtering error covariance matrix

Calculation：

For *n* = 1, 2, …, the calculation is shown below:

$$\alpha_{n}=\mathbf{d}_{n}-\mathbf{b}_{n}(\widehat{\mathbf{w}}_{n|n-1},\mathbf{v}_{n},\mathbf{u}_{n})$$

$$\widehat{\mathbf{w}}_{n+1|n}=\widehat{\mathbf{w}}_{n|n}$$

$$\mathbf{P}_{n|n}=\mathbf{P}_{n|n-1}-\mathbf{G}_{n}\mathbf{B}_{n}\mathbf{P}_{n|n-1}$$

$$\mathbf{P}_{n+1|n}=\mathbf{P}_{n|n}+\mathbf{Q}_{w,n}$$

Initialization：

$P_{1|0}=\delta^{-1}\mathbf{I}$, $\delta$ was a small normal number, and $\mathbf{I}$ was the unit matrix

To use the EKF to finish the supervised learning task, we needed to retrain the nonlinear part of equation (17) and use the first order Taylor expansion to linearize the measurement equation of equation (17). $\mathbf{b}(\mathbf{w}_{n},\mathbf{v}_{n},\mathbf{u}_{n})$ was the only nonlinear source. The following equation was used to approximate equation (17):

$\mathbf{d}_{n}\approx\mathbf{B}_{n}\mathbf{w}_{n}+\mathbf{\upsilon}_{n}$ (20)

where $\mathbf{B}_{n}$ was the measurement matrix of the linearized model $p\times s$. During the linearization process, the partial derivative of *p* outputs on $s$ weights in the recurrent network was calculated to obtain the matrix:

$\mathbf{B}=\left[\begin{matrix} \frac{\partial b_{1}}{\partial w_{1}} & \frac{\partial b_{1}}{\partial w_{2}} & \cdots& \frac{\partial b_{1}}{\partial w_{s}} \\ \frac{\partial b_{2}}{\partial w_{1}} & \frac{\partial b_{2}}{\partial w_{2}} & \cdots& \frac{\partial b_{2}}{\partial w_{s}} \\ \vdots& \vdots& \vdots& \vdots\\ \frac{\partial b_{p}}{\partial w_{1}} & \frac{\partial b_{p}}{\partial w_{2}} & \cdots& \frac{\partial b_{p}}{\partial w_{s}} \end{matrix}\right]$ (21)

The dimension of matrix $\mathbf{B}$ was $p\times s$. The dimension of the weight vector $\mathbf{w}$ is $s$; thus, the matrix product $\mathbf{Bw}$ was a $p\times1$ vector, which matched with the dimension of the observed value $\mathbf{d}$. In equation (21), $n$ was omitted to simplify the expression. In the equation, $b_{i}$, $i=1,2,\cdots,p$ was recorded as the $i$^th^ element of the vector function $b(\mathbf{w}_{n},\mathbf{v}_{n},\mathbf{u}_{n})$. The partial derivative of the right-end item of equation (20) was evaluated at $\mathbf{w}_{n}=\hat{\mathbf{w}}_{n|n-1}$. Equation (21) was calculated using the algorithm described in RTRL.

The above algorithms are cited from the book *Neural Networks and Learning Machines, third ed.,* written by Simon Haykin (Prentice Hall). All analyses were conducted using MATLAB R2012a (The MathWorks, USA, 2012).

Table S1. The numbers of new confirmed cases of 8 types of infectious diseases per month

| **Time** | **Measles** | **Influenza** | **Rubella** | **Mumps** | **Hepatitis A** | **Hepatitis E** | **Typhoid fever and paratyphoid fever** | **Bacillary and amoebic dysentery** |
| --- | --- | --- | --- | --- | --- | --- | --- | --- |
| Jan 2004 | 2711 | 2477 | 576 | 9315 | 6402 | 1429 | 2594 | 13232 |
| Feb 2004 | 3780 | 6236 | 858 | 6640 | 8058 | 1813 | 3148 | 12768 |
| Mar 2004 | 8192 | 11691 | 2899 | 19623 | 9052 | 2227 | 3473 | 16967 |
| Apr 2004 | 11076 | 10311 | 6377 | 29343 | 8473 | 1752 | 3948 | 24102 |
| May 2004 | 10449 | 3062 | 5273 | 30155 | 8891 | 1541 | 4869 | 44174 |
| Jun 2004 | 8358 | 2276 | 3666 | 32510 | 8404 | 1467 | 5106 | 64973 |
| Jul 2004 | 5950 | 1656 | 1321 | 24388 | 8337 | 1249 | 6031 | 92058 |
| Aug 2004 | 3008 | 1952 | 555 | 10961 | 8950 | 1188 | 6070 | 91568 |
| Sep 2004 | 2641 | 4195 | 454 | 8988 | 7898 | 1022 | 5035 | 64514 |
| Oct 2004 | 3810 | 1932 | 524 | 14337 | 7792 | 973 | 4288 | 36438 |
| Nov 2004 | 5233 | 1934 | 733 | 20033 | 6736 | 1052 | 3030 | 22926 |
| Dec 2004 | 5341 | 1774 | 779 | 20526 | 4594 | 731 | 1740 | 14159 |
| Jan 2005 | 8236 | 2146 | 1101 | 23649 | 5726 | 1539 | 2080 | 13173 |
| Feb 2005 | 7448 | 1179 | 705 | 12266 | 1604 | 4534 | 1652 | 10722 |
| Mar 2005 | 17936 | 4653 | 2744 | 18890 | 6923 | 2190 | 2132 | 15165 |
| Apr 2005 | 28812 | 8723 | 6358 | 37034 | 6202 | 1838 | 2472 | 20608 |
| May 2005 | 22802 | 3961 | 5536 | 36777 | 6481 | 1327 | 3444 | 38441 |
| Jun 2005 | 15267 | 3213 | 4166 | 39950 | 6286 | 1183 | 3940 | 59688 |
| Jul 2005 | 7936 | 1258 | 1458 | 29478 | 6367 | 1038 | 4172 | 85757 |
| Aug 2005 | 3594 | 1314 | 575 | 13262 | 6847 | 1052 | 4169 | 78728 |
| Sep 2005 | 2237 | 2487 | 512 | 11839 | 6496 | 942 | 3608 | 60256 |
| Oct 2005 | 2301 | 4504 | 661 | 18731 | 6759 | 997 | 3308 | 38531 |
| Nov 2005 | 3027 | 7378 | 858 | 25665 | 6331 | 1045 | 2494 | 22693 |
| Dec 2005 | 3540 | 4856 | 772 | 23594 | 4397 | 786 | 1225 | 12779 |
| Jan 2006 | 5857 | 3355 | 1096 | 19033 | 4550 | 1704 | 1147 | 10919 |
| Feb 2006 | 5960 | 4777 | 1111 | 10655 | 4936 | 2219 | 1324 | 10633 |
| Mar 2006 | 12831 | 15828 | 5166 | 23521 | 6157 | 2768 | 1709 | 15478 |
| Apr 2006 | 18641 | 12271 | 8501 | 32423 | 5637 | 2116 | 2214 | 21182 |
| May 2006 | 18505 | 5188 | 7622 | 35068 | 5931 | 1818 | 2617 | 35296 |
| Jun 2006 | 12203 | 3947 | 6304 | 38361 | 5949 | 1490 | 2635 | 49440 |
| Jul 2006 | 7512 | 3042 | 2119 | 26669 | 5755 | 1228 | 3116 | 67506 |
| Aug 2006 | 4249 | 1811 | 715 | 13121 | 6596 | 1207 | 3144 | 75180 |
| Sep 2006 | 2822 | 1387 | 653 | 11545 | 5803 | 1042 | 2667 | 56810 |
| Oct 2006 | 2825 | 1475 | 1170 | 18239 | 6218 | 1179 | 2453 | 43506 |
| Nov 2006 | 3527 | 1652 | 1153 | 20008 | 6011 | 1213 | 1941 | 23504 |
| Dec 2006 | 4670 | 2824 | 1409 | 22754 | 5124 | 1023 | 1019 | 13678 |
| Jan 2007 | 8912 | 6340 | 2541 | 22743 | 5675 | 2183 | 1062 | 12366 |
| Feb 2007 | 9263 | 1990 | 2512 | 12428 | 4426 | 2039 | 807 | 10425 |
| Mar 2007 | 11210 | 3581 | 6895 | 12457 | 6406 | 2616 | 1272 | 13944 |
| Apr 2007 | 16333 | 3652 | 19508 | 24877 | 6013 | 2176 | 1489 | 18681 |
| May 2007 | 18074 | 2609 | 20406 | 29161 | 6513 | 1842 | 1970 | 34143 |
| Jun 2007 | 12567 | 3603 | 11748 | 31912 | 6717 | 1643 | 2520 | 49275 |
| Jul 2007 | 10086 | 1837 | 3710 | 25793 | 7342 | 1571 | 2703 | 59525 |
| Aug 2007 | 6263 | 1483 | 1209 | 13730 | 8266 | 1612 | 2891 | 63194 |
| Sep 2007 | 3382 | 2077 | 844 | 11495 | 7465 | 1334 | 1964 | 45995 |
| Oct 2007 | 3181 | 2144 | 1265 | 17731 | 7328 | 1306 | 1819 | 29099 |
| Nov 2007 | 4168 | 2786 | 1490 | 23604 | 6464 | 1292 | 1235 | 18014 |
| Dec 2007 | 5584 | 4332 | 2618 | 26770 | 4520 | 963 | 696 | 13321 |
| Jan 2008 | 13235 | 4366 | 3571 | 26544 | 4578 | 1843 | 723 | 10834 |
| Feb 2008 | 13925 | 2635 | 2736 | 13103 | 3828 | 1871 | 570 | 9617 |
| Mar 2008 | 20491 | 7854 | 15988 | 20557 | 5505 | 2726 | 909 | 13681 |
| Apr 2008 | 21740 | 3822 | 27612 | 31548 | 5449 | 2112 | 1118 | 18395 |
| May 2008 | 24908 | 2985 | 38633 | 49564 | 5824 | 1786 | 1518 | 33287 |
| Jun 2008 | 14079 | 2021 | 18393 | 42111 | 5284 | 1425 | 1827 | 40068 |
| Jul 2008 | 7912 | 2332 | 5537 | 32509 | 5249 | 1305 | 2008 | 47550 |
| Aug 2008 | 3906 | 2631 | 1695 | 15733 | 5084 | 1226 | 2074 | 46595 |
| Sep 2008 | 2063 | 3050 | 1000 | 13165 | 4399 | 1061 | 1638 | 36672 |
| Oct 2008 | 2160 | 2729 | 1374 | 18867 | 4166 | 1096 | 1486 | 27168 |
| Nov 2008 | 3092 | 3236 | 1628 | 22647 | 3703 | 1097 | 1067 | 16837 |
| Dec 2008 | 3930 | 4031 | 2187 | 24478 | 2983 | 977 | 703 | 11818 |
| Jan 2009 | 4483 | 3526 | 1766 | 19550 | 3100 | 1930 | 651 | 9440 |
| Feb 2009 | 5426 | 4505 | 2258 | 12049 | 3651 | 2444 | 852 | 9955 |
| Mar 2009 | 8838 | 8597 | 9326 | 28095 | 4350 | 2730 | 1146 | 13497 |
| Apr 2009 | 13189 | 6814 | 19427 | 41725 | 4173 | 2048 | 1305 | 17832 |
| May 2009 | 9806 | 7461 | 17607 | 46363 | 4319 | 1735 | 1637 | 28873 |
| Jun 2009 | 5056 | 8841 | 10854 | 45417 | 4294 | 1487 | 1796 | 33313 |
| Jul 2009 | 2520 | 7383 | 3610 | 32838 | 4164 | 1338 | 2167 | 40077 |
| Aug 2009 | 1059 | 16180 | 1271 | 15296 | 4104 | 1424 | 2073 | 40645 |
| Sep 2009 | 633 | 41807 | 1013 | 13371 | 3587 | 1157 | 1839 | 31963 |
| Oct 2009 | 429 | 25478 | 954 | 14090 | 3099 | 1195 | 1543 | 22177 |
| Nov 2009 | 366 | 43640 | 861 | 13833 | 2597 | 1237 | 1122 | 13034 |
| Dec 2009 | 656 | 24149 | 913 | 16702 | 2403 | 1550 | 807 | 10745 |
| Jan 2010 | 1302 | 10501 | 1063 | 18088 | 2496 | 2288 | 648 | 8976 |
| Feb 2010 | 2577 | 6340 | 982 | 11143 | 2016 | 2304 | 556 | 8156 |
| Mar 2010 | 5163 | 8484 | 2880 | 13149 | 2792 | 3076 | 858 | 11107 |
| Apr 2010 | 8875 | 5896 | 7454 | 23364 | 2898 | 2567 | 975 | 14237 |
| May 2010 | 11476 | 3697 | 13381 | 38371 | 3022 | 2180 | 1235 | 23167 |
| Jun 2010 | 3909 | 2617 | 8526 | 43256 | 2971 | 1719 | 1239 | 28627 |
| Jul 2010 | 1727 | 2644 | 3400 | 35711 | 3210 | 1584 | 1613 | 37117 |
| Aug 2010 | 1144 | 3922 | 1289 | 19902 | 3726 | 1461 | 1868 | 41093 |
| Sep 2010 | 798 | 4875 | 998 | 14950 | 3374 | 1421 | 1631 | 33527 |
| Oct 2010 | 404 | 4106 | 828 | 18354 | 3339 | 1468 | 1578 | 21762 |
| Nov 2010 | 312 | 5267 | 963 | 27362 | 2870 | 1684 | 985 | 13935 |
| Dec 2010 | 472 | 6153 | 1353 | 35282 | 2563 | 1930 | 855 | 10544 |
| Jan 2011 | 561 | 5854 | 1342 | 31202 | 2198 | 2342 | 567 | 7959 |
| Feb 2011 | 944 | 5991 | 1594 | 16417 | 2109 | 2930 | 529 | 7925 |
| Mar 2011 | 1469 | 7095 | 5606 | 25402 | 2878 | 4178 | 685 | 11011 |
| Apr 2011 | 2185 | 5408 | 14852 | 41104 | 2657 | 3270 | 821 | 13538 |
| May 2011 | 1955 | 4117 | 18241 | 55927 | 2705 | 2459 | 1063 | 22549 |
| Jun 2011 | 1130 | 2951 | 12783 | 59623 | 2676 | 2118 | 1160 | 31396 |
| Jul 2011 | 564 | 2589 | 4204 | 53224 | 2842 | 2010 | 1328 | 36570 |
| Aug 2011 | 321 | 3170 | 1483 | 27724 | 3060 | 2116 | 1487 | 37453 |
| Sep 2011 | 217 | 4203 | 1028 | 19825 | 2704 | 1871 | 1216 | 27134 |
| Oct 2011 | 167 | 5548 | 1173 | 29288 | 2706 | 1872 | 1174 | 18719 |
| Nov 2011 | 144 | 7242 | 1453 | 41088 | 2645 | 2060 | 1041 | 13388 |
| Dec 2011 | 286 | 11965 | 1790 | 53561 | 2276 | 1974 | 727 | 10288 |
| Jan 2012 | 245 | 10836 | 1251 | 38786 | 1841 | 1969 | 685 | 8276 |
| Feb 2012 | 231 | 19125 | 1638 | 21183 | 2206 | 3180 | 726 | 8307 |
| Mar 2012 | 347 | 19887 | 4641 | 35640 | 2230 | 3557 | 850 | 10585 |
| Apr 2012 | 572 | 9385 | 9546 | 50823 | 2016 | 2662 | 878 | 12940 |
| May 2012 | 700 | 8312 | 9757 | 67982 | 2151 | 2354 | 1185 | 20432 |
| Jun 2012 | 676 | 6163 | 5959 | 71255 | 1953 | 1889 | 1267 | 25228 |
| Jul 2012 | 647 | 7104 | 2292 | 55443 | 2170 | 1843 | 1296 | 30724 |
| Aug 2012 | 554 | 6424 | 1019 | 26604 | 2333 | 1906 | 1347 | 29668 |
| Sep 2012 | 312 | 6307 | 852 | 19290 | 2018 | 1737 | 1174 | 22636 |
| Oct 2012 | 445 | 7236 | 896 | 25717 | 1906 | 1763 | 1065 | 17669 |
| Nov 2012 | 557 | 8911 | 1104 | 30497 | 1974 | 1959 | 845 | 11955 |
| Dec 2012 | 897 | 12450 | 1126 | 36298 | 1596 | 2083 | 705 | 9144 |
| Jan 2013 | 1749 | 15766 | 1251 | 37245 | 1653 | 3076 | 662 | 8323 |
| Feb 2013 | 2673 | 9413 | 984 | 21715 | 1396 | 3145 | 628 | 7673 |
| Mar 2013 | 4188 | 10815 | 2133 | 24834 | 1884 | 3628 | 932 | 10195 |
| Apr 2013 | 4945 | 10746 | 3067 | 37883 | 1811 | 2772 | 1069 | 11713 |
| May 2013 | 4711 | 8550 | 3600 | 45277 | 1797 | 2241 | 1309 | 16611 |
| Jun 2013 | 2770 | 6216 | 2198 | 43232 | 1701 | 1794 | 1595 | 23037 |
| Jul 2013 | 2248 | 5270 | 1137 | 34360 | 2045 | 1874 | 1758 | 27722 |
| Aug 2013 | 1755 | 6156 | 690 | 18612 | 2070 | 1899 | 1594 | 27497 |
| Sep 2013 | 956 | 8786 | 583 | 14415 | 2051 | 1723 | 1418 | 20844 |
| Oct 2013 | 549 | 9670 | 605 | 14903 | 1949 | 1764 | 1279 | 16196 |
| Nov 2013 | 445 | 12880 | 594 | 17388 | 1998 | 1960 | 998 | 10745 |
| Dec 2013 | 657 | 25605 | 738 | 17895 | 1889 | 2026 | 894 | 8113 |
| Jan 2014 | 2245 | 37334 | 670 | 14251 | 1632 | 2655 | 783 | 6586 |
| Feb 2014 | 4719 | 25306 | 791 | 8994 | 1792 | 3018 | 621 | 6276 |
| Mar 2014 | 8702 | 26038 | 1922 | 14205 | 2028 | 3140 | 859 | 8333 |
| Apr 2014 | 10766 | 12406 | 2235 | 18344 | 1857 | 2510 | 954 | 10457 |
| May 2014 | 9578 | 13153 | 2202 | 24246 | 1922 | 2052 | 1226 | 14456 |
| Jun 2014 | 6152 | 19616 | 1389 | 23785 | 1996 | 1836 | 1574 | 19810 |
| Jul 2014 | 3862 | 13694 | 731 | 19316 | 2180 | 1958 | 1610 | 22158 |
| Aug 2014 | 2196 | 10923 | 449 | 12962 | 2393 | 2054 | 1632 | 20355 |
| Sep 2014 | 994 | 9009 | 324 | 12176 | 2461 | 1908 | 1634 | 16720 |
| Oct 2014 | 969 | 8672 | 335 | 12475 | 2644 | 1849 | 1264 | 12725 |
| Nov 2014 | 855 | 13123 | 337 | 13208 | 2597 | 1883 | 878 | 8632 |
| Dec 2014 | 1590 | 26259 | 408 | 13538 | 2467 | 2125 | 733 | 7077 |
| Jan 2015 | 3655 | 23080 | 735 | 14152 | 2419 | 2893 | 684 | 6619 |
| Feb 2015 | 4743 | 13962 | 654 | 9202 | 1540 | 2301 | 583 | 5780 |
| Mar 2015 | 6496 | 19653 | 1246 | 11222 | 2054 | 3032 | 830 | 7781 |
| Apr 2015 | 8129 | 13950 | 1937 | 16382 | 1769 | 2668 | 851 | 9362 |
| May 2015 | 7268 | 14608 | 1663 | 21241 | 1756 | 2165 | 1178 | 14080 |
| Jun 2015 | 4313 | 34584 | 761 | 23292 | 1743 | 1956 | 1401 | 17699 |
| Jul 2015 | 2843 | 19650 | 319 | 19569 | 2008 | 1991 | 1290 | 19088 |
| Aug 2015 | 1705 | 11780 | 182 | 13311 | 1907 | 1931 | 1192 | 18106 |
| Sep 2015 | 722 | 9008 | 148 | 12413 | 1940 | 1792 | 1114 | 14665 |
| Oct 2015 | 540 | 9422 | 138 | 12873 | 1897 | 1982 | 1003 | 11673 |
| Nov 2015 | 730 | 10979 | 158 | 13920 | 1826 | 2131 | 832 | 7862 |
| Dec 2015 | 1217 | 15047 | 192 | 15256 | 1808 | 2327 | 679 | 6202 |
| Jan 2016 | 2041 | 23333 | 288 | 12941 | 1800 | 2342 | 671 | 5193 |
| Feb 2016 | 2971 | 31602 | 224 | 8077 | 1721 | 2363 | 580 | 5258 |
| Mar 2016 | 5437 | 76498 | 706 | 10823 | 2198 | 3401 | 893 | 6314 |
| Apr 2016 | 5488 | 47530 | 1183 | 13837 | 2063 | 2927 | 881 | 7756 |
| May 2016 | 4317 | 17901 | 1155 | 17889 | 1802 | 2478 | 1043 | 11942 |
| Jun 2016 | 2850 | 10011 | 785 | 20096 | 1628 | 2263 | 1070 | 14607 |
| Jul 2016 | 1761 | 7479 | 315 | 18231 | 1723 | 2198 | 1248 | 16701 |
| Aug 2016 | 929 | 7859 | 140 | 13507 | 1823 | 2260 | 1344 | 18347 |
| Sep 2016 | 414 | 11212 | 124 | 12829 | 1695 | 2031 | 1190 | 14607 |
| Oct 2016 | 264 | 13165 | 141 | 13269 | 1654 | 1884 | 949 | 11002 |
| Nov 2016 | 336 | 20800 | 162 | 16073 | 1875 | 2168 | 821 | 6699 |
| Dec 2016 | 462 | 38313 | 251 | 18511 | 1884 | 2356 | 739 | 5953 |
| Jan 2017 | 611 | 30109 | 179 | 14865 | 1438 | 2150 | 651 | 5130 |
| Feb 2017 | 628 | 22998 | 123 | 10208 | 1616 | 2588 | 567 | 5227 |
| Mar 2017 | 1087 | 30519 | 268 | 16469 | 1978 | 3352 | 715 | 5884 |
| Apr 2017 | 992 | 23260 | 296 | 22554 | 1684 | 2714 | 722 | 7174 |
| May 2017 | 865 | 19085 | 331 | 31840 | 1606 | 2671 | 982 | 10975 |
| Jun 2017 | 46 | 22313 | 195 | 33458 | 1609 | 2567 | 1242 | 14169 |
| Jul 2017 | 438 | 57694 | 148 | 26431 | 1556 | 2410 | 1323 | 16316 |
| Aug 2017 | 379 | 41625 | 104 | 17691 | 1774 | 2608 | 1365 | 15314 |
| Sep 2017 | 305 | 27467 | 97 | 16142 | 1787 | 2269 | 1158 | 11078 |
| Oct 2017 | 245 | 16049 | 100 | 15936 | 1530 | 1927 | 1001 | 8537 |
| Nov 2017 | 257 | 27731 | 126 | 22922 | 1597 | 2286 | 805 | 6109 |
| Dec 2017 | 260 | 121800 | 193 | 26280 | 1428 | 2302 | 770 | 4954 |
| Jan 2018 | 377 | 273949 | 117 | 21540 | 1329 | 2762 | 860 | 4219 |
| Feb 2018 | 417 | 139738 | 72 | 11238 | 1054 | 2291 | 507 | 4009 |
| Mar 2018 | 609 | 74086 | 250 | 14858 | 1422 | 3377 | 694 | 5336 |
| Apr 2018 | 587 | 28592 | 472 | 23015 | 1368 | 2807 | 731 | 6321 |
| May 2018 | 665 | 22980 | 766 | 31707 | 1437 | 2632 | 1014 | 9657 |
| Jun 2018 | 522 | 16635 | 431 | 33314 | 1609 | 2567 | 1242 | 14169 |
| Jul 2018 | 327 | 14544 | 281 | 25731 | 1587 | 2386 | 1278 | 12786 |
| Aug 2018 | 231 | 12325 | 199 | 16801 | 1640 | 2368 | 1466 | 12447 |
| Sep 2018 | 172 | 13188 | 289 | 15657 | 1376 | 2023 | 1222 | 9579 |
| Oct 2018 | 168 | 14852 | 284 | 17443 | 1314 | 1896 | 1133 | 7249 |
| Nov 2018 | 223 | 26960 | 446 | 23472 | 1420 | 2264 | 907 | 5255 |
| Dec 2018 | 185 | 130442 | 786 | 26717 | 1367 | 2335 | 685 | 4523 |
